# Sleep biomarkers of seasonal vulnerability in major depressive episodes: a clinical study using actigraphy and polysomnography

**DOI:** 10.1016/j.ijchp.2025.100595

**Published:** 2025-06-13

**Authors:** Julia MARUANI, Lily VISSOUZE, Marine AMBAR AKKAOUI, Feriel ZEHANI, Justine FRIJA, Michel LEJOYEUX, Marie-Pia d’ORTHO, Pierre A. GEOFFROY

**Affiliations:** a Département de psychiatrie et d'addictologie, AP-HP, GHU Paris Nord, DMU Neurosciences, Hôpital Bichat - Claude Bernard, F-75018 Paris, France; b Université Paris Cité, NeuroDiderot, Inserm, FHU I2-D2, F-75019 Paris, France; c Centre ChronoS, GHU Paris - Psychiatrie & Neurosciences, 1 rue Cabanis, 75014 Paris, France; d Centre Psychiatrique d'Orientation et d'Accueil (CPOA), GHU Paris Psychiatrie et Neurosciences, Hôpital Sainte Anne, Paris, France; e SAMU 75, SAMU psychiatrique, AP-HP, Hôpital Necker, 75015 Paris, France; f Explorations Fonctionnelles et Centre du Sommeil-Département de Physiologie Clinique, AP-HP, GHU Paris Nord, DMU DREAM, Hôpital Bichat - Claude Bernard, F-75018, Paris, France; g CNRS UPR 3212 & Strasbourg University, Institute for Cellular and Integrative Neurosciences, F–67000, Strasbourg, France

**Keywords:** Depressive disorders, Seasonal affective disorder, Polysomnography, Actigraphy, Seasonal pattern

## Abstract

Seasonal affective disorder or major depressive episode (MDE) with winter seasonal pattern is a severe form of recurrent depressive disorder persisting for at least 40 % of the year. This seasonal vulnerability may involve specific chronobiological and sleep regulation disturbances, however, actigraphy and polysomnography studies reported conflicting and inconclusive findings. In this context, we hypothesized that individuals with MDE who exhibit higher vulnerability to seasonal changes display distinct chronobiological and sleep regulation disturbances. We investigated how sleep and circadian rhythms, assessed through subjective markers (*N* = 254) and objective actigraphic (*N* = 96) and polysomnographic markers (*N* = 54)—varied in individuals with MDE according to their vulnerability to seasonal changes. To conduct this dimensional approach, that may be closer to the pathophysiology, we assessed the seasonal vulnerability using the Global Seasonality Score (GSS) from the Seasonal Patterns Assessment Questionnaire (SPAQ). The results revealed that greater seasonal vulnerability was correlated to increased excessive daytime sleepiness (Epworth scale), (*r* = 0.218, *p* < 0.001), shorter REM sleep latency (*r* = -0.381, *p* = 0.005), (even when controlling for age using the Kupfer index (*r* = -0.452, *p* < 0.001)), and shorter N1 sleep latency (*r* = -0.411, *p* = 0.002). The regression model retained only the polysomnographic markers—N1 sleep latency, REM sleep latency, Kupfer Index—independently and significantly associated to the GSS. This study confirms a link between seasonal vulnerability and shortened REM sleep latency, emphasizing that light and the photoperiod play a pivotal role in regulating the systems involved in REM sleep dysregulation in depressive disorders.

## Highlights

• The vulnerability to seasons independently correlates with shorter latencies for both REM and N1 sleep.
• The shorter the REM sleep latency was, the higher was the vulnerability to seasons, even after controlling for age with the Kupfer Index.
• The light and photoperiod seem to play a pivotal role in regulating the systems involved in REM sleep dysregulation in depressive disorders.

## Introduction

Depressive disorders, projected by the WHO to become the leading cause of disability by 2030, encompass a broad spectrum of prevalent and severe conditions, affecting up to 20 % of the general population (Bakish, 2001; Malhi et al., 2005). These disorders represent a critical public health concern, significantly impairing psychosocial functioning and quality of life, and are closely linked to a high prevalence of suicide (WHO, 2004). Currently, the diagnosis of a Major Depressive Episode (MDE) relies entirely on clinical evaluation and established diagnostic criteria from the American Psychiatric Association (APA) (Association AP. Diagnostic & Statistical Manual of Mental Disorders: Dsm-5. Amer Psychiatric Pub Incorporated;, 2013. 947 p, s. d.). Despite substantial international research aimed at identifying biomarkers for depression, none have yet been successfully translated into clinical practice for diagnosis, prognosis, or therapeutic prediction (Malhi et al., 2005). One major obstacle to progress in this area is the substantial heterogeneity of depressive disorders. Consequently, it is crucial to better define and differentiate subtypes of depressive disorders through the use of biomarkers. Sleep disturbances, observed in >90 % of individuals with MDE (Geoffroy et al., 2018), hold particular promise as potential biomarkers for refining the classification of depressive subtypes (Geoffroy & Gottlieb, 2020; Geoffroy & Palagini, 2021). Objective sleep-related markers identified via polysomnography include increased rapid eye movement (REM) sleep density, reduced REM latency, and decreased Slow Wave Sleep (SWS) duration (Benca et al., 1992; Bertrand et al., 2021; Kupfer, 1976; Kupfer et al., 1986a; Palagini et al., 2013). Some depressive subtypes appear more homogeneous due to their distinct sleep and circadian rhythm abnormalities (Solelhac et al., 2023). For instance, Seasonal Affective Disorder (SAD), called also MDE with winter Seasonal Pattern, is a subtype characterized by recurrent MDE occurring in the autumn and winter, triggered by reduced daylight hours (e.g. photoperiod), and fully remitting during the spring or summer (Rosenthal, 1984). Recent studies in MDE with winter Seasonal Pattern reported several sleep subjective abnormalities such as hypersomnolence or excessive daytime sleepiness (Rosenthal, 1984; Wescott et al., 2022, 2023) and objective abnormalities using both actigraphy and polysomnography (PSG) (Bertrand et al., 2021; Wescott et al., 2022, 2023). Actigraphy studies comparing patients with MDE with winter Seasonal Pattern and non-depressed controls also indicated more specific sleep and circadian abnormalities (Wescott et al., 2022, 2023). However, these studies highlighted various sleep and circadian disruptions in seasonal depression, with some reporting delayed sleep/wake timing, others observing advanced sleep/wake timing, and some noting hypersomnia while others did not (Wescott et al., 2022, 2023; Winkler et al., 2005). The PSG studies also highlighted various sleep disruptions in seasonal depression. Some reported alterations of the total sleep time (TST) and SWS amounts (Palagini et al., 2013), while others observed increases in REM sleep and shortening of REM sleep latency in patients with SAD (Brunner et al., 1996; Koorengevel et al., 2002). However, a recent meta-analysis emphasized increases in REM sleep and shortening of REM sleep latency as consistent trait markers of SAD (Bertrand et al., 2021). This meta-analyse did not confirm significant alterations of TST and SWS amounts in patients with SAD. In spite of methodological differences and small sample sizes, the meta-analyses benefited from stricter inclusion criteria and controlled medication use, enhancing result reliability.

Discrepancies observed with previous studies may arise from the categorical approach, which may not fully capture the underlying pathophysiology. A dimensional approach, accounting for varying levels of vulnerability to seasonal, may provide a more accurate framework. This approach is best assessed with the Seasonal Patterns Assessment Questionnaire (SPAQ) (N. et al., 1984), a widely used tool validated for measuring seasonality in unipolar and bipolar disorders (Reynaud et al., 2021). Seasonal variations appear to influence mood and behavior, with SAD potentially representing the pathological end of a continuum of the vulnerability to season regarding mood changes with photoperiods. These dimensional aspects likely reflect the underlying biological processes as a continuum of seasonal effects' severity and no biomarkers have yet been identified to better understand the effects of season on MDE using this more dimensional perspective, closer to biological mechanisms. In this context, we hypothesized that individuals with MDE who exhibited a higher vulnerability to seasons, potentially extending to SAD, showed more specific sleep alterations, as well as more specific chronobiological disturbances. Therefore, we investigated how subjective and objective sleep and circadian markers assessed through actigraphy and PSG, varied in patients with MDE based on their vulnerability to seasonal changes as measured by the SPAQ (Reynaud et al., 2021).

## Method

### Study design

Patients were recruited during their visit or hospitalization for MDE at the Department of Psychiatry and Addictology at Bichat Claude Bernard Hospital in Paris. Patients were included from November 2019 to September 2024. Patients' medical histories were collected (treatments, recurrences, progression) and, with questionnaires, current MDE characteristics, comorbidities and seasonal profile. Data were collected during routine MDE assessments following DSM-5 criteria (*Association AP. Diagnostic & Statistical Manual of Mental Disorders: Dsm-5. Amer Psychiatric Pub Incorporated; 2013**. 947 p*, s. d.; Geoffroy et al., 2013). The seasonal pattern (SP) used to classify patients as having or not having winter seasonal depression was based on the DSM-5 criteria as previously detailed in the study of Geoffroy et al., 2013 and according to the Global Seasonality Score (GSS) of the SPAQ, which has been validated in French and in populations with mood disorders (Reynaud et al., 2021). Sleep and circadian rhythms markers were evaluated by subjective (self-report questionnaires) and objective (actigraphy for at least 14 days and PSG) measures. In the context of routine care, some patients were asked to wear an actigraph (MotionWatch 8 ®) on the wrist of their non-dominant hand for at least 14 consecutives days, as recommended by the American Academy of Sleep Medicine (AASM) (Smith et al., 2018) and some of them received at least one night of PSG recording at Bichat Hospital Sleep Centre for evaluation of a sleep disorder between November 2019 and June 2024. The detailed clinical protocol is described in previous work (Mauries et al., 2023).

Participants were eligible if they were aged between 18 and 65, had a MDE according to DSM-5 criteria (Diagnostic & statistical manual of mental disorders, 2013), were affiliated to a social security scheme and had signed a non-objection form for the use of their data (as required by French law).

Exclusion criteria included people who were unable to give informed consent due to urgent situations or comprehension difficulties, individuals who worked in shifts or had irregular work hours in the year preceding inclusion and had traveled across more than two time zones in the month before inclusion.

Patients were included in the Som-Psy cohort (PI: PA Geoffroy, ethically approved (N °CER-2020–56) by the “Comité d’Évaluation de l’Éthique des projets de Recherche Biomédicale (CEERB) Paris Nord” (Institutional Review Board -IRB 00,006,477- of the HUPNVS, Université Paris 7, AP-HP)).

### Sociodemographic, sleep and psychiatric clinical parameters

Sociodemographic characteristics such as gender, age and body mass index (BMI) were collected. Clinical parameters were assessed, including number of depressive episodes, number of hospitalizations, treatments taken and the severity of depressive symptoms (using the hetero-questionnaire Montgomery-Asberg Depression Rating Scale (MADRS),(Montgomery & Asberg, 1979)).

In addition, other self-report questionnaires have been used to assess sleep symptoms and characterize circadian rhythms. These include the Pittsburgh Sleep Quality Index (PSQI) (Buysse et al., 1989), which assesses sleep quality; the Epworth Sleepiness Scale (ESS) which assesses sleepiness (Gonçalves et al., 2023); the Insomnia Severity Index (ISI) which assesses insomnia (Morin et al., 2011); and the 19-item Morningness–Eveningness Questionnaire (MEQ) which assesses the circadian typology (Taillard et al., 2004).

### Seasonality assessment

Vulnerability to season was assessed through the Seasonal Patterns Assessment Questionnaire (SPAQ), validated in French by our team, that allows for this dimensional assessment and is widely used in clinical studies, showing good validity metrics, including in patients with mood disorders (N. et al., 1984, (Reynaud et al., 2021). The Global Seasonality Score (GSS) of the SPAQ was used to assess seasonality, more precisely the seasonal change across 6 dimensions, namely sleep length, social activity, mood, weight, appetite and energy level (Reynaud et al., 2021). The degree of change of the 6 dimensions were assessed by a single item each scored from 0 (no change) to 4 (extremely marked change). The 6 scores were summed up to calculate the general seasonality score (GSS), which thus ranges from 0 (no seasonality) to 24 (extreme seasonality).

### Objective sleep and circadian rhythms measures

#### Actigraphy

The biomarkers explored by the Motion Watch 8 actigraphy include 6 measures of sleep profile 1) time in bed, 2) total night sleep time (TST), 3) sleep onset latency, 4) wake after sleep onset or nocturnal awakenings (WASO) indicating the period of wake time in sleep period, 5) sleep fragmentation index indicating the degree of fragmentation of the sleep period, and 6) sleep efficiency (SE) representing the total sleep time (TST) divided by the time in bed (in percentage). The circadian rhythms biomarkers measured by the Motion Watch 8 actigraphy (CamNTech) over the entire 14-day period include: 1) 4 measures of activity: the actigraphy markers of phase with the L5 onset which is the onset of the 5 hours the least active and the M10 onset which is the onset of the 10 hours the most active. The M10 average measures activity during the most active 10-hours period and reflects how active the wake periods are, the L5 average measures activity during the least active 5-hours period (usually nighttime), reflects resting levels during the night and a lower L5 indicates less more restful sleep; 2) 2 measures of variability (inter-daily stability (IS) (day-to-day stability of the sleep-wake rhythm) and intradaily variability (IV) (intra-day sleep-wake rhythm variability)) (Wescott et al., 2022, 2023) 3) one measure of rhythm amplitude: the relative amplitude is the difference between M10 and L5 in the average 24-hours pattern, normalized by their sum; higher relative amplitudes therefore indicate a more robust 24-hours rest-activity rhythm, reflecting both relatively lower activity during the night and higher activity when awake.

#### Polysomnography

The biomarkers explored by the PSG (V-PSG; Morpheus by Micromed; Noxturnal by ResMed; Alice 5, Philips-Respironics, Suresnes, France) include 20 measures of sleep architecture: N1, N2, N3, REM sleep latency and duration (min), Time in bed (min), TTS (min), N1, N2, N3, REM %TTS, Kupfer Index (KI), Slow-Wave Sleep (SWS) Duration (min), Duration of the first N3 sleep (min), WASO (min), sleep efficiency, Total awakening index, Apnea/hypopnea index. The Kupfer index (KI), proposed as a biomarker for depression (Ansseau et al., 1987), was calculated as the sum of age and REM latency (Kupfer et al., 1982) and was explored because several studies have found that normal values of REM sleep latency decrease with age (Ansseau et al., 1987; Kupfer et al., 1985, 1986b).

### Statistics

The relationships between the vulnerability to season (GSS) and the sleep, circadian and psychiatrics quantitative variables were studied with a correlation matrix using the Pearson (r) or Spearman (Rho) correlation coefficient. Each categorical variable was described with a number and percentage, whilst each continuous variable was described with a mean and standard deviation (SD). The normality of the continuous variables’ distribution was assessed with visual inspection of Q-Q plots and with the statistical test of normality (Shapiro-Wilk test *p* > 0.05). Benjamini and Hochberg corrections were applied instead of the Bonferroni correction, which tends to be more stringent and risks excluding false negatives. The Bonferroni correction, while effective in controlling the overall type I error rate, is often too conservative, increasing the risk of failing to detect true positives (false negatives) (Korthauer et al., 2019). The evolution of GSS as a function of the selected variables was modelled with linear regression to assess independent associations of variables previously found to be significant in univariate analyses. Additionally, before conducting these regression analyses, the absence of collinearity among the variables was verified to ensure the stability of coefficients and the validity of conclusions drawn from these analyses. All statistical analyses were performed using JAMOVI software (version 2.4.14), where a p-value of <0.05 was used as the significance threshold.

### Sensitivity analysis

Since the inclusions comprised patients with both unipolar and bipolar depressive disorders, subgroup analyses were performed to explore potential differences in seasonality-related outcomes. We conducted additional correlation analyses within these two subpopulations, using sociodemographic data and scores from the various assessment questionnaires. We also carried out correlation tests between seasonality and PSG/actigraphy results in patients with unipolar disorders (MDD). These latter analyses were not performed in the group with bipolar disorders (BD) due to the very limited number of patients who underwent actigraphy and/or PSG. The results of these additional analyses are presented in supplementary Tables 1 to 8.

## Results

### Clinical and socio-demographic characteristics

A total of 254 patients with MDE were included in this study (Table 1.1), consisting of 163 women and 91 men, with a mean age of 41.6 years, (Table 1.1). Among the 254 patients, 182 (71.65 %) had an MDE as part of a unipolar disorder, while 40 (15.75 %) had an MDE in the context of a bipolar disorder. On average, the patients both with unipolar and bipolar depression, had experienced 1.84 hospitalizations and 4.02 depressive episodes. Among them, 96 wore an actigraph, and 54 underwent one night of PSG, (Table 1.2, Table 1.3 respectively). The number of patients taking medications such as antidepressants, anxiolytics, hypnotics, mood stabilizers, or antipsychotics is also provided in Tables 1.1, 1.2, and 1.3. All 254 participants completed the SPAQ, with a mean GSS of 10.5 (range: 0 to 24).

**Table 1.1:** Socio-demographic and clinical characteristics of patients with major depressive episode (MDE) who were included in the study. *N* = 254.

Variables		Means (± SE) or n ( %)
Gender		
	Female	163 (64.2 %)
	Male	91 (35.83 %)
Polarity	Unipolar	182 (71.65 %)
	Bipolar	40 (15.75 %)
Age (years)		41.6 ± 14.7
BMI (kg/m²)		26.6 ± 6.81
Number of hospitalizations		1.84 ± 3.33
Number of depressive episodes		4.02 ± 5.49
MADRS		23.7 ± 8.13
Global Score of Seasonality (GSS)		10.5 ± 6.1
Insomnia (ISI)		18.4 ± 5.16
Sleep quality (PSQI)		12.3 ± 3.84
Sleepiness (ESS)		9.37 ± 5.53
Chronotype (MEQ)		46.5 ± 11.2
Antidepressants	Yes	130 (69.15 %)
	No	58 (30.85 %)
Antipsychotics	Yes	16 (8.33 %)
	No	176 (91.67 %)
Anxiolytics	Yes	72 (37.89 %)
	No	118 (62.11 %)
Hypnotics	Yes	67 (35.08 %)
	No	124 (64.92 %)
Mood stabilizers	Yes	51 (26.84 %)
	No	139 (73.16 %)

BMI: Body Mass Index. MADRS: Montgomery-Asberg Depression Rating Scale. MEQ: Morningness-Eveningness Questionnaire.

**Table 1.2:** Socio-demographic and clinical characteristics of patients with MDE who underwent actigraphy. *N* = 96.

Variables		Means (± SE) or n
Gender		
Female		65 (67.7 %)
Male		31 (32.3 %)
Age (years)		38.5 ± 13.6
BMI (kg/m²)		26.7 ± 7.18
Number of hospitalizations		1.49 ± 2.2
Number of depressive episodes		3.67 ± 4.73
MADRS		25.2 ± 7.47
Global Score of Seasonality (GSS)		10.9 ± 6.11
Insomnia (ISI)		18 ± 5.18
Sleep quality (PSQI)		12.2 ± 4.17
Sleepiness (ESS)		9.37 ± 5.62
Chronotype (MEQ)		46.6 ± 11.7
Antidepressants	Yes	68 (72.34 %)
	No	26 (27.66 %)
Antipsychotics	Yes	8 (8.51 %)
	No	86 (91.49 %)
Anxiolytics	Yes	34 (36.17 %)
	No	60 (63.83 %)
Hypnotics	Yes	36 (42.86 %)
	No	58 (69.05 %)
Mood stabilizers	Yes	31 (32.63 %)
	No	64 (67.37 %)

BMI: Body Mass Index, MADRS: Montgomery-Asberg Depression Rating Scale. ISI: Insomnia Severity Index. PSQI: Pittsburgh Sleep Quality Index. ESS: Epworth Sleepiness Scale. MEQ: Morningness-Eveningness Questionnaire.

**Table 1.3:** Socio-demographic and clinical characteristics of patients with MDE who underwent polysomnography. *N* = 54.

Variables		Means (± SE) or n
Gender		
Female		31 (57.41 %)
Male		23 (42.59 %)
Age (years)		43.8 ± 12.7
BMI (kg/m²)		27.1 ± 6.86
Number of hospitalizations		0.619 ± 0.973
Number of depressive episodes		4.59 ± 6.84
MADRS		21.3 ± 5.83
Global Score of Seasonality (GSS)		10.5 ± 6.11
Insomnia (ISI)		18 ± 5.34
Sleep quality (PSQI)		12.9 ± 3.8
Sleepiness (ESS)		9.74 ± 5.6
Chronotype (MEQ)		46.8 ± 11.1
Antidepressants	Yes	16 (48.48 %)
	No	17 (51.52 %)
Antipsychotics	Yes	1 (3.03 %)
	No	32 (96.97 %)
Anxiolytics	Yes	10 (31.25 %)
	No	22 (68.75 %)
Hypnotics	Yes	7 (21.88 %)
	No	25 (78.13 %)
Mood stabilizers	Yes	4 (11.76 %)
	No	30 (88.24 %)

BMI: Body Mass Index, MADRS: Montgomery-Asberg Depression Rating Scale. ISI: Insomnia Severity Index. PSQI: Pittsburgh Sleep Quality Index. ESS: Epworth Sleepiness Scale. MEQ: Morningness-Eveningness Questionnaire.

The Insomnia Severity Index (ISI) had a mean score of 18.4, indicating moderate insomnia, while the Pittsburgh Sleep Quality Index (PSQI) had a mean score of 12.3, reflecting impaired sleep quality. The Epworth Sleepiness Scale (ESS) had a mean score of 9.37, indicating sleepiness but not excessive sleepiness. The Morningness–Eveningness Questionnaire (MEQ) had a mean score of 46.5, indicating an intermediate chronotype (neither morning nor evening type). The Montgomery–Åsberg Depression Rating Scale (MADRS) showed a mean score of 23.7, indicating moderately severe depressive symptoms.

### Correlations between the vulnerability to season (GSS) and sociodemographic and psychiatric characteristics

No significant correlations were found between the GSS obtained from the SPAQ and the following factors: age, BMI, number of depressive episodes, number of hospitalizations and severity of depression (as assessed by the MADRS total score), (Table 2.1).

**Table 2.1:** Correlation matrix showing the relationships between Global Seasonality Score (GSS) assessed with the SPAQ and socio-demographic and clinical characteristics of patients with major depressive episode (MDE).

Variables	Rho Spearman GSS	*p*
Age (years)	−0.115	0.077
BMI (kg/m²)	0.009	0.892
Number of depressive episodes	−0.110	0.155
Number of hospitalizations	−0.014	0.858
MADRS score	0.132	0.074

SPAQ GSS: Seasonal Pattern Assessment Questionnaire, Global Seasonality Score.

BMI: Body Mass Index, MADRS: Montgomery-Asberg Depression Rating Scale.

### Correlations between the vulnerability to season (GSS) and subjective sleep and circadian variables

Excessive daytime sleepiness, assessed with the ESS, significantly correlated with the GSS, showing a correlation of *r* = 0.218, *p* < 0.001 (Table 2.2), with no other correlations.

**Table 2.2:** Correlation matrix showing the relationships between Global Seasonality Score (GSS) and subjective sleep variables of patients with major depressive episode (MDE).

Variables	Rho Spearman GSS	*p*
Insomnia severity (ISI total score)	0.063	0.352
Sleep quality (PSQI total score)	0.074	0.279
Excessive Daytime Sleepiness (ESS)	**0.218**	**<0.001**
Chronotype (MEQ)	−0.003	0.971

SPAQ GSS: Seasonal Pattern Assessment Questionnaire, Global Seasonality Score.

ISI: Insomnia Severity Index. PSQI: Pittsburgh Sleep Quality Index. ESS: Epworth Sleepiness Scale. MEQ: Morningness-Eveningness Questionnaire. Bolded results indicate p-values that remained significant after Benjamini-Hochberg statistical correction.

### Correlations between the vulnerability to season (GSS) and objective sleep and circadian actigraphic variables

No significant correlations were observed (Table 2.3).

**Table 2.3:** Correlation matrix showing the relationships between Global Seasonality Score (GSS) and actigraphic sleep variables.

Variables	Rho Spearman GSS	*p*
Time in bed	0.103	0.314
Total Sleep Time (TST)	0.232	0.092
WASO	−0.140	0.323
Sleep efficiency ( %)	0.033	0.751
Sleep latency	0.108	0.293
Fragmentation Index	−0.086	0.400
L5 average	0.151	0.141
L5 onset	−0.037	0.718
M10 average	0.107	0.301
M10 onset	−0.028	0.786
RA Relative Amplitude	−0.097	0.348
IS Inter-daily Stability	−0.007	0.948
IV Intra-daily variability	−0.040	0.696

SPAQ GSS: Seasonal Pattern Assessment Questionnaire, Global Seasonality Score.

L5= least five, M10 = most ten, TST = total night sleep time, WASO = wake after sleep onset.

### Correlations between the vulnerability to season (GSS) and objective sleep and circadian polysomnographic variables

A significant negative correlation was found between the GSS and the average N1 sleep latency (*r* = −0.411, *p* = 0.002), the average REM sleep latency (*r* = −0.381, *p* = 0.005), and the Kupfer Index (*r* = −0.452, *p* < 0.001), (Table 2.4).

**Table 2.4:** Correlation matrix showing the relationships between Global Seasonality Score (GSS) and polysomnographic sleep variables.

Variables	Rho Spearman GSS	*p*
N1 stage latency (min)	**−0.411**	**0.002**
N1 stage duration (min)	−0.073	0.598
N1 % TST	−0.103	0.456
N2 stage latency (min)	−0.216	0.132
N2 stage duration (min)	0.153	0.270
N2 % TST	−0.072	0.601
N3 stage latency (min)	−0.210	0.254
N3 stage duration (min)	0.184	0.184
N3 % TST	0.100	0.467
REM sleep latency	**−0.381**	**0.005**
Kupfer Index	**−0.452**	**<0.001**
REM stage duration (min)	0.225	0.101
REM % TST	0.131	0.348
Total Slow-Wave Sleep Duration (min)	−0.065	0.747
Duration of the first N3 stage (min)	−0.008	0.960
TST (min)	0.232	0.092
Time in bed	−0.108	0.571
WASO (min)	−0.140	0.323
Sleep efficiency ( %)	0.190	0.168
Total awakening index.	0.111	0.623
Apnea/hypopnea index	−0.228	0.098

Bolded results indicate p-values that remained significant after Benjamini-Hochberg statistical correction.

SPAQ GSS: Seasonal Pattern Assessment Questionnaire, Global Seasonality Score.

TST = total night sleep time, WASO = wake after sleep onset, REM: Rapid Eye Movement.

N1: first stage of non-rapid eye movement (NREM) sleep. N2: second stage of non-rapid eye movement (NREM) sleep. N3: final stage of non-rapid eye movement (NREM) sleep, also known as slow-wave sleep (SWS).

### Linear regression model of GSS evolution based on selected variables from univariate analyses

Weconducted regression analyses to identify which variables, initially correlated with seasonal vulnerability in univariate analyses, remained independently and significantly associated in a multivariate context. We built two models, one including the REM sleep latency, the other the Kupfer Index, as these two variables were correlated. Both models also included objective polysomnographic markers and subjective markers. The aim was to determine whether any objective sleep markers were independently associated with seasonal vulnerability, beyond the influence of subjective markers. The regression model ultimately found that the three objective polysomnographic markers—N1 sleep latency (i.e., the time required to fall asleep), REM sleep latency, and the Kupfer Index—independently and significantly were associated to vulnerability to seasonality (N1 sleep latency *p* = 0.012 (Table 3.1) and 0.037 (Table 3.2), REM sleep latency *p* = 0.007 Table 3.1, and *p* = 0.003, Table 3.2). These regression models were adjusted for medications that impact sleep: antidepressants were found to influence REM sleep latency, while hypnotics affected ESS scores. The use of antidepressants did not alter the significance of the associations between REM sleep latency (*p* = 0.043), the Kupfer index (*p* = 0.032), or N1 sleep latency (*p* = 0.044) and GSS. Similarly, the use of hypnotics did not change the significance of the associations between REM sleep latency (*p* = 0.035), the Kupfer index (*p* = 0.022), and GSS. However, it did affect the association between N1 sleep latency and GSS, rendering it non-significant (*p* = 0.085).

**Table 3.1:** Linear regression model of the GSS evolution from the SPAQ based on selected variables from univariate analyses.

Predictor of vulnerability to season (GSS)	Estimation (± SE)	*p*
N1 stage latency (min)	−0.0804 (±0.03076)	**0.012**
REM stage latency (min)	−0.0276 (±0.00977)	**0.007**
Excessive daytime sleepiness (ESS)	0.0345 (±0.14425)	0.812

REM: Rapid Eye Movement. ESS: Epworth Sleepiness Scale. Bold values indicate a statistically significant difference with a p-value <0.05. Estimates represent unstandardized coefficients (B) ± standard error.

**Table 3.2:** Linear regression model of GSS evolution from the SPAQ based on selected variables from univariate analyses.

Predictor of vulnerability to season (GSS)	Estimation (± SE)	*p*
N1 stage latency (min)	−0.0650 (±0.03021)	**0.037**
Kupfer Index	−0.0292 (±0.00943)	**0.003**
Excessive daytime sleepiness (ESS)	0.0298 (±0.14640)	0.840

ESS: Epworth Sleepiness Scale. Bold value indicates a statistically significant difference with a p-value <0.05. Estimates represent unstandardized coefficients (B) ± standard error.

### Sensitivity analyses

Out of the 254 patients included in the study, we had polarity data for 222 of them. Among these, 40 were diagnosed with bipolar depression and 182 with unipolar depression. We had complete qualitative data (sociodemographic and questionnaire responses) for 180 patients with MDD and for all 40 patients with BD. Among the patients with MDD, 82 underwent actigraphy and 34 underwent PSG. Only 3 patients with BD underwent both actigraphy and PSG.

#### Correlation analysis

We conducted correlation analyses between the GSS and sociodemographic and clinical data specifically in the 182 patients with MDD (Supplementary Tables 1 and 2) and in the 40 patients with BD specifically (Supplementary Tables 3 and 4). A significant positive correlation was found between the MADRS score and the GSS in the patients with MDD (Rho=0.224, *p* = 0.010) (Supplementary Table 1), but not in the patients with BD (Rho = 0.036, *p* = 0.837) (Supplementary Table 3). Significant positive correlations were confirmed between the GSS and the ESS in the patients with MDD (Rho=0.208, *p* = 0.005) (Supplementary Table 2) and also confirmed for patients with BD (Rho=0.345, *p* = 0.029) (Supplementary Table 4). No other significant correlations were found between the GSS and the other sociodemographic or clinical data in the patients with MDD and BD consistent with the results from the analysis of the entire patient sample. Regarding actigraphy data, correlation analyses with the GSS were conducted on the data from the 82 patients with MDD who underwent this test; no significant correlation was found, consistent with the analysis of the entire patient sample (Supplementary Table 5). Finally, correlation analyses between the GSS and the data from the 34 patients with MDD who underwent PSG confirmed a significant negative correlation with N1 latency (Rho=−0.354, *p* = 0.047), REM sleep latency (Rho=−0.407, *p* = 0.023), and the Kupfer index (Rho=−0.558, *p* = 0.002) (Supplementary Table 6). Only 3 patients with BD underwent both actigraphy and PSG, which did not justify the use of statistical tests on these data.

#### Linear regression analysis

Finally, we conducted linear regression analyses based on the variables that were found to be significant in the correlation tests for the patients with MDD; in the first model, we included the scores obtained on the ESS and MADRS, as well as the N1 latency and the Kupfer index. We observed that only the Kupfer index significantly and independently predicted the GSS (*p* = 0.013) (Supplementary Table 7). We then included the scores obtained on the ESS and MADRS, as well as the REM sleep latency and N1 latency, in a second regression model and observed that only REM sleep latency significantly and independently predicted the GSS (*p* = 0.010) (Supplementary Table 8). These results are consistent with the analysis of the entire patient sample, except that we did not confirm N1 latency as a significant predictor of GSS in patient with MDD. These regression models were adjusted for medications taken by patients with MDD which impact their sleep: antidepressants (which influence MADRS scores and REM sleep latency), anxiolytics (which affect both MADRS and ESS scores), and hypnotics (which influence ESS scores). The use of antidepressants did not alter the significance of the associations between REM sleep latency (*p* = 0.013) or the Kupfer index (*p* = 0.020) and GSS. Similarly, the use of anxiolytics did not affect the significance of the associations between REM sleep latency (*p* = 0.011) or the Kupfer index (*p* = 0.012) and GSS. Finally, the use of hypnotics did not modify the significance of the associations between either REM sleep latency (*p* = 0.050) or the Kupfer index (*p* = 0.046) and GSS.

## Discussion

Our study observed for the first time that individuals with depression who exhibited a higher vulnerability to seasonal changes (GSS), showed shorter N1 sleep latency and shorter REM sleep latency or lower Kupfer Index (to account and correct for the age by considering the sum of REM sleep latency and age). These findings validate and extend previous research demonstrating a strong association between SAD—considered the type of depression most severely affected by seasonal changes—and shorter REM sleep latency (Bertrand et al., 2021). Indeed, in a recent meta-analysis, shortening of REM sleep latency was identified a trait marker of SAD, as REM sleep latency was significantly decreased in patients with remitted SAD, with a similar trend observed during the acute phase (Bertrand et al., 2021). However, in contrast to this meta-analysis, our study did not confirm an increase in REM sleep duration in depressed patients with seasonal vulnerability. This suggests that a general vulnerability to seasonal changes may not involve exactly the same mechanistic pathways as those underlying SAD. Additionally, this meta-analysis showed that vulnerability to seasonal changes in patients with MDE was not associated with alterations in TST or SWS amounts. Thus, our findings confirm, using a dimensional approach, both categorical findings from the meta-analysis by Bertrand et al. (2021) and the meta-analysis by Baglioni et al. (2016), which found no alterations in TST or SWS in patients with SAD (Baglioni et al., 2016; Bertrand et al., 2021). Interestingly, our study did not observe correlations between actigraphic markers and vulnerability to seasonal changes in patient with MDE. This stands in contrast to previous actigraphy studies comparing patients with MDE with winter Seasonal Pattern to non-depressed controls. Those studies highlighted various sleep and circadian disruptions in seasonal depression compared to non-depressed controls, with some reporting delayed sleep/wake timing, others advanced sleep/wake timing, and some observing hypersomnia while others did not (Wescott et al., 2022, 2023; Winkler et al., 2005). From a dimensional perspective, our findings further suggest that a general vulnerability to seasonal changes may involve distinct mechanistic pathways compared to the pathophysiological processes underpinning SAD as a discrete disorder. SAD represents a more severe subtype of depression, characterized by recurring annual depressive episodes and symptoms persisting for at least 40 % of the year. Recent efforts had increasingly focused on identifying objective biomarkers for SAD. Moving beyond the categorical approach, which delineates a dichotomy between MDE with and without seasonal patterns, our study, using a more refined approach, suggests that sleep polysomnographic markers—rather than actigraphic markers—may offer deeper insights into the biological mechanisms associated with seasonal vulnerability. Finally, our study found that individuals with depression who exhibited a higher vulnerability to seasonal changes showed excessive daytime sleepiness but not independently of abnormalities in REM sleep or N1 sleep. This finding aligns with previous studies, which showed that the majority of patients with SAD did not endorse abnormal levels of daytime sleepiness, making hypersomnolence a poor general characterization of MDE with seasonal pattern (Wescott et al., 2023). These results contribute to the ongoing debate regarding the nature of hypersomnolence in seasonal depression. While we observed a significant correlation between ESS and seasonal vulnerability, this association appears to be mediated by specific REM and N1 sleep alterations, suggesting that ESS may reflect a downstream consequence of circadian misalignment or disrupted sleep architecture, rather than representing an independent clinical feature.

Our study is the first to establish a significant link between seasonal vulnerability and shortened REM sleep latency. Interestingly, our sensitivity analyses, which explored sleep biomarkers separately in patients with MDD and BD —given their substantial differences in genetics, symptoms, pathophysiology, medication, treatment, and biomarkers— confirmed positive correlation between ESS and GSS in bipolar MDE, confirmed positive correlation between ESS and GSS and negative correlation between N1 sleep latency, REM sleep latency and GSS in unipolar MDE and revealed that, in unipolar MDE, PSG REM sleep latency was the only biomarker that significantly and independently predicted the GSS.

More than 90 % of patients with depression have sleep and circadian disturbances. The most consistent EEG changes associated with depression include a decrease in REM sleep latency, an increase in total REM sleep time, higher REM density, and a reduction in SWS (Palagini et al., 2013). These REM sleep alterations, especially shortened REM latency or low Kupfer Index are considered biological markers of depression and even indicators of vulnerability to the disorder (Ansseau et al., 1987; Kupfer et al., 1982, 1985, 1986b). Studies have shown that these changes persist even during remission and also seem predictive of conversion, recurrence, and therapeutic responses (Palagini et al., 2013). Despite this, no study has yet successfully identified or fully understood the specific characteristics or pathophysiological factors underlying shortened REM sleep latency or a decreased Kupfer index observed in depression. While, two previous studies suggested that Shortened REM sleep latency correlates positively with illness severity (Kupfer & Foster, 1972; Spiker et al., 1978), other research has demonstrated that factors such as age can also influence REM sleep latency (Lauer et al., 1991; Palagini et al., 2013). Our study is the first to identify seasonal vulnerability as a dimensional factor present across all types of depression, implicating it in the pathophysiological mechanisms associated with shortened REM sleep latency and decreased Kupfer Index. For the first time, our findings emphasize the role of light—specifically, the impact of seasonal changes in light duration and illumination (e.g. photoperiod)—in the dysregulation of REM sleep observed in depression. This is not surprising, as light plays a crucial role in regulating all the systems involved in REM sleep dysregulation (Palagini et al., 2013). Four main mechanisms appear to be involved in the regulation of REM sleep latency (Palagini et al., 2013). The cholinergic-aminergic imbalance hypothesis suggests that the shortened REM sleep latency in depression may result from an overactivity of central cholinergic neurotransmission. Another explanation involves a deficiency in process "S", which could lead to earlier REM sleep onset, though our findings did not show the expected reduction in SWS. A third hypothesis proposes circadian rhythm disturbances, specifically a phase advance, to account for REM sleep abnormalities, a theory supported by our observation of reduced N1 sleep latency. Finally, the last hypothesis involves the hypocretin (orexin) system, highlighting the role of orexin in stabilizing wakefulness and REM sleep transitions. Light exerts powerful biological effects on mood regulation (Maruani & Geoffroy, 2022) and plays a key role in mood in modulating REM sleep dysregulation (Maruani & Geoffroy, 2022). Light influences mood by aligning circadian rhythms, modulating sleep homeostasis (which can affect SWS), and enhancing alertness and emotional regulation. Light also activates key brain regions associated with wakefulness, such as monoaminergic systems, the thalamus, and hypothalamic orexin pathways, all involved in REM sleep regulation. Our study provides additional insights into the mechanisms of REM sleep dysregulation in depression suggesting that light and photoperiod play a pivotal role in regulating the systems underlying REM sleep dysregulation in depressive disorders. MDE are heterogeneous disorders, and patients may exhibit individual and specific abnormalities in light-signaling pathways. Some individuals may display distinct chronobiological anomalies, such as disruptions in clock genes, while others may present different disturbances in light-signaling pathways, such as orexinergic dysfunctions or homeostatic dysregulation. In our study, the depressed group appeared to align more closely with the chronobiological hypothesis of seasonal vulnerability, suggesting a phase advance in circadian rhythms (even though we did not identify circadian markers through subjective measures or actigraphy, which are less precise than physiological measures of circadian timing, such as Dim Light Melatonin Onset (DLMO) or core body temperature nadir). This finding contrasts with the sleep homeostasis (process S) disruption hypothesis, as vulnerability to seasonality was associated with shorter N1 and REM sleep latencies rather than deficits in slow-wave sleep.

Some limitations of this study should be acknowledged. The main limitation of our study was the small number of included patients with PSG, underscoring the need to validate these findings with a larger and independent sample. Moreover, we exclusively enrolled depressed patients and did not incorporate a healthy control group. Including such a group would have enabled us to determine whether individuals without depression exhibit a similar short REM sleep latency, suggesting that short REM sleep latency might explain difficulties in seasonal adaptation independently of depression. Short REM Sleep latency may not be specific to depression but rather to seasonal changes. Furthermore, it would be interesting to explore REM sleep latency in these patients during remission to determine whether short REM sleep latency is a state or trait marker. The literature suggests that short REM sleep latency is more likely a trait marker, possibly even an endophenotype, which, according to the results of our study, may signal vulnerability to photoperiod-related seasonal changes.

To conclude, in depression, seasonal vulnerability may influence REM sleep latency, as outlined by the previously mentioned mechanisms and Kupfer index may serve as a marker of photoperiod-related seasonal changes. In the future, it would be interesting to explore whether the antidepressant effect of light therapy could normalize REM sleep latency in depression. Indeed, it would be valuable to investigate whether REM sleep latency can serve as a predictive marker for the response to light therapy, which is the reference treatment for Seasonal Affective Disorder (SAD). If this were found to be the case, it could lead to more personalized medicine, potentially offering light therapy specifically to patients with MDE with shortened REM sleep latency.

## Statement

During the preparation of this work, we used generative AI technologies to edit the English text

## Declaration of competing interest

The authors declare no conflicts of interest related to this study.

## Appendix Supplementary materials

Image, application 1
