# Supplementary material for: Sleep biomarkers of seasonal vulnerability in major depressive episodes: a clinical study using actigraphy and polysomnography
Source: Int J Clin Health Psychol. 2025 Jun 13;25(2):100595. doi: 10.1016/j.ijchp.2025.100595 (PMC12205580; doi:10.1016/j.ijchp.2025.100595)
Supplement: Supplementary file 1 [file mmc1.docx]

**Supplementary table** 1**.** Correlation matrix showing the relationships between Global Seasonality Score (GSS) assessed with the SPAQ and socio-demographic and clinical characteristics of patients with unipolar major depressive episode (MDE). N=182

| **Variables** | **Rho Spearman GSS** | ***p*** |
| --- | --- | --- |
| Age (years) | -0.112 | 0.137 |
| BMI (kg/m²) | -0.009 | 0.907 |
| Number of depressive episodes | -0.077 | 0.363 |
| Number of hospitalizations | 0.026 | 0.765 |
| MADRS score | 0.224 | **0.010** |

*SPAQ GSS: Seasonal Pattern Assessment Questionnaire, Global Seasonality Score.*

*BMI: Body Mass Index, MADRS: Montgomery-Asberg Depression Rating Scale. Bold value indicates a statistically significant difference with a p-value less than 0.05.*

**Supplementary table 2.** Correlation matrix showing the relationships between Global Seasonality Score (GSS) and subjective sleep variables of patients with unipolar major depressive episode (MDE). N=182

| **Variables** | **Rho Spearman GSS** | ***p*** |
| --- | --- | --- |
| Insomnia severity (ISI total score) | 0.105 | 0.170 |
| Sleep quality (PSQI total score) | 0.102 | 0.178 |
| Excessive Daytime Sleepiness (ESS) | **0.208** | **0.005** |
| Chronotype (Horne & Ostberg) | -0.022 | 0.771 |

*SPAQ GSS: Seasonal Pattern Assessment Questionnaire, Global Seasonality Score.*

*ISI: Insomnia Severity Index. PSQI: Pittsburgh Sleep Quality Index. ESS: Epworth Sleepiness Scale. Bold value indicates a statistically significant difference with a p-value less than 0.05.*

**Supplementary table** **3.** Correlation matrix showing the relationships between Global Seasonality Score (GSS) assessed with the SPAQ and socio-demographic and clinical characteristics of patients with bipolar major depressive episode (MDE). N=40

| **Variables** | **Rho Spearman GSS** | ***p*** |
| --- | --- | --- |
| Age (years) | -0.005 | 0.976 |
| BMI (kg/m²) | 0.143 | 0.386 |
| Number of depressive episodes | -0.068 | 0.726 |
| Number of hospitalizations | -0.085 | 0.623 |
| MADRS score | 0.036 | 0.837 |

*SPAQ GSS: Seasonal Pattern Assessment Questionnaire, Global Seasonality Score.*

*BMI: Body Mass Index, MADRS: Montgomery-Asberg Depression Rating Scale.*

**Supplementary table 4.** Correlation matrix showing the relationships between Global Seasonality Score (GSS) and subjective sleep variables of patients with bipolar major depressive episode (MDE). N=40

| **Variables** | **Rho Spearman GSS** | ***p*** |
| --- | --- | --- |
| Insomnia severity (ISI total score) | -0.089 | 0.635 |
| Sleep quality (PSQI total score) | -0.102 | 0.542 |
| Excessive Daytime Sleepiness (ESS) | **0.345** | **0.029** |
| Chronotype (Horne & Ostberg) | 0.086 | 0.656 |

*SPAQ GSS: Seasonal Pattern Assessment Questionnaire, Global Seasonality Score.*

*ISI: Insomnia Severity Index. PSQI: Pittsburgh Sleep Quality Index. ESS: Epworth Sleepiness Scale. Bold value indicates a statistically significant difference with a p-value less than 0.05.*

**Supplementary table 5.** Correlation matrix showing the relationships between Global Seasonality Score (GSS) and actigraphic sleep variables among patients with unipolar depression. N=82.

| **Variables** | **Rho Spearman GSS** | ***p*** |
| --- | --- | --- |
| Time in bed | -0.176 | 0.379 |
| Total Sleep Time (TST) | 0.032 | 0.860 |
| WASO | -0.275 | 0.134 |
| Sleep efficiency (%) | 0.029 | 0.802 |
| Sleep latency | 0.136 | 0.246 |
| Fragmentation Index | -0.067 | 0.566 |
| L5 average | 0.181 | 0.124 |
| L5 onset | -0.014 | 0.905 |
| M10 average | 0.126 | 0.284 |
| M10 onset | -0.035 | 0.766 |
| RA Relative Amplitude | -0.127 | 0.279 |
| IS Inter-daily Stability | -0.013 | 0.910 |
| IV Intra-daily variability | -0.032 | 0.786 |

*SPAQ GSS: Seasonal Pattern Assessment Questionnaire, Global Seasonality Score.*

*L5= least five, M10 = most ten, TST = total night sleep time, WASO = wake after sleep onset*

**Supplementary table 6**. Correlation matrix showing the relationships between Global Seasonality Score (GSS) and polysomnographic sleep variables among patients with unipolar depression. N=34.

| **Variables** | **Rho Spearman GSS** | ***p*** |
| --- | --- | --- |
| N1 stage latency (min) | **-0.354** | **0.047** |
| N1 stage duration (min) | -0.159 | 0.385 |
| N1% TST | -0.152 | 0.407 |
| N2 stage latency (min) | -0.221 | 0.231 |
| N2 stage duration (min) | 0.173 | 0.344 |
| N2% TST | -0.023 | 0.899 |
| N3 stage latency (min) | -0.310 | 0.095 |
| N3 stage duration (min) | 0.008 | 0.967 |
| N3% TST | 0.058 | 0.752 |
| REM sleep latency | **-0.407** | **0.023** |
| Kupfer Index | **-0.558** | **0.002** |
| REM stage duration (min) | 0.141 | 0.441 |
| REM % TST | 0.183 | 0.316 |
| Total Slow-Wave Sleep Duration (min) | -0.136 | 0.527 |
| Duration of the first N3 stage (min) | -0.163 | 0.457 |
| TST (min) | 0.032 | 0.860 |
| Time in bed | -0.176 | 0.379 |
| WASO (min) | -0.275 | 0.134 |
| Sleep efficiency (%) | 0.155 | 0.397 |
| Total awakening index. | 0.063 | 0.785 |
| Apnea/hypopnea index | -0.145 | 0.428 |

*Bold values indicate a statistically significant difference with a p-value less than 0.05.*

*SPAQ GSS: Seasonal Pattern Assessment Questionnaire, Global Seasonality Score.*

*TST = total night sleep time, WASO = wake after sleep onset, REM: Rapid Eye Movement.*

*N1: first stage of non-rapid eye movement (NREM) sleep. N2: second stage of non-rapid eye movement (NREM) sleep. N3: final stage of non-rapid eye movement (NREM) sleep, also known as slow-wave sleep (SWS).*

| **Supplementary table 7**. Linear regression model of the GSS evolution from the SPAQ based on selected variables from univariate analyses among the 34 patients with unipolar depression who underwent polysomnography. |
| --- |
| \| **Predictor of vulnerability to season (GSS)** \| **Estimation (± SE)** \| ***p*** \| \| --- \| --- \| --- \| \| N1 stage latency (min) \| -0.0565 (±0.0390) \| 0.167 \| \| REM stage latency (min) \| -0.0342 (±0.0116) \| **0.010** \| \| Excessive daytime sleepiness (ESS) \| 0.3810 (±0.2165) \| 0.098 \| \| MADRS \| 0.4359 (±0.2645) \| 0.119 \| |

| **Supplementary table 8.** Linear regression model of the GSS evolution from the SPAQ based on selected variables from univariate analyses among the 34 patients with unipolar depression who underwent polysomnography. |
| --- |
| \| **Predictor of vulnerability to season (GSS)** \| **Estimation (± SE)** \| ***p*** \| \| --- \| --- \| --- \| \| N1 stage latency (min) \| -0.0390 (±0.0378) \| 0.318 \| \| Kupfer index \| -0.0318 (±0.0112) \| **0.013** \| \| Excessive daytime sleepiness (ESS) \| 0.1670 (±0.2456) \| 0.507 \| \| MADRS \| 0.3006 (±0.2651) \| 0.275 \| |
